# Future physicians and tobacco: an online survey of the habits, beliefs and knowledge base of medical students at a Canadian University

**DOI:** 10.1186/1617-9625-11-9

**Published:** 2013-04-04

**Authors:** Amanda J Vanderhoek, Fadi Hammal, Alyssa Chappell, T Cameron Wild, Tobias Raupach, Barry A Finegan

**Affiliations:** 1 Department of Anesthesiology and Pain Medicine, University of Alberta, Edmonton, Canada; 2 School of Public Health, University of Alberta, Edmonton, Canada; 3 Department of Cardiology and Pneumology, University Hospital Göttingen, Göttingen, Germany

**Keywords:** Medical education, Tobacco cessation, Waterpipe, Medical students

## Abstract

**Background:**

Little is known about the knowledge and attitudes towards tobacco use among medical students in Canada. Our objectives were to estimate the prevalence of tobacco use among medical students, assess their perceived level of education about tobacco addiction management and their preparedness to address tobacco use with their future patients.

**Methods:**

A cross-sectional online survey was administered to University of Alberta undergraduate medical school trainees. The 32-question survey addressed student demographics, tobacco use, knowledge and attitudes around tobacco and waterpipe smoking, tobacco education received in medical school, as well as knowledge and competency regarding tobacco cessation interventions.

**Results:**

Of 681 polled students, 301 completed the survey. Current (defined as “use within the last 30 days”) cigarette, cigar/cigarillo and waterpipe smoking prevalence was 3.3%, 6% and 6%, respectively. One third of the respondents had ever smoked a cigarette, but 41% had tried cigars/cigarillos and 40% had smoked a waterpipe at some time in the past. Students reported moderate levels of education on a variety of tobacco-related subjects but were well-informed on the role of tobacco in disease causation. The majority of students in their final two years of training felt competent to provide tobacco cessation interventions, but only 10% definitively agreed that they had received enough training in this area.

**Conclusions:**

Waterpipe exposure/current use was surprisingly high among this sample of medical students, a population well educated about the role of tobacco in disease causation. The majority of respondents appeared to be adequately prepared to manage tobacco addiction but education could be improved, particularly training in behavioral modification techniques used in tobacco use cessation.

## Background

Physicians play a crucial role in preventing disease and promoting a healthy lifestyle [1]. The willingness of physicians to engage in providing prevention counseling is influenced by many factors, especially, their training [2], their own personal habits and their self-confidence in doing so effectively [3,4]. This “personal-clinical relationship” has been well validated among Canadian and US physicians [4,5] and is especially relevant in the area of tobacco control, as non-smoking physicians are more likely to report encouraging cessation among their patients than those who smoke [4]. A similar trend is found among medical students, where healthy personal practices are found to equate with engagement in preventive counseling and appreciation of importance of this role as part of their professional responsibility [6]. A combination of didactic and interactive training during medical school can improve knowledge, attitude, and counseling skills regarding tobacco cessation and other forms of behavioral change [7,8]. There is very limited information about the prevalence of smoking among Canadian medical school students and their perceived level of education and preparedness to address tobacco use with their patients [9]. There are no data on waterpipe use in medical students, a popular emerging method of smoking that is frequently thought of as less harmful than cigarettes [10].

In this cross-sectional survey of University of Alberta undergraduate medical students, we sought to report on their smoking status, what their opinions were regarding the risks of waterpipe smoking, the extent of their knowledge about the role of tobacco in disease causation and their understanding of the relative effectiveness of currently used tobacco cessation interventions. Finally, we requested they provide their views on the education they received about tobacco addiction management, their self-perceived capacity/competency to treat patients with tobacco addiction and the role of physician leadership in advancing tobacco control and management.

## Methods

Students in the undergraduate Doctor of Medicine program in the Faculty of Medicine and Dentistry at the University of Alberta, Edmonton, Canada, were invited to participate in a cross-sectional, anonymous on-line survey. The questionnaire was available on-line for a six-week period in 2012. During that time, we tracked the completion of questionnaires, noting the date and time of survey completion. Four email reminders were sent out during the course of the survey, each of which contained a summary of the invitation letter and link to the questionnaire.

The undergraduate program spans four years, beginning with two years of preclinical education. In the third year, students begin their clinical education, which consists of two years of clerkship in a variety of medical specialties. During the fourth year of their education, students are given three months of protected time to undergo 10–15 weeks of electives, interview for residency positions and prepare for graduation. The pre-clinical medical curriculum at the University of Alberta is based on system blocks focusing on small group learning. Tobacco related education is integrated into each system block with no special block devoted solely to tobacco. Therefore it was difficult to estimate how many hours of tobacco education medical students receive throughout their undergraduate medical education. There were approximately 170 students enrolled in each year of the program for the 2011/12 school year. All 681 students in the graduating classes of 2012 to 2015 (46.4% female) were eligible to participate in the survey.

### Development of the survey instrument

The questions were developed with guidance of previously used questionnaires on tobacco and education among medical students [11,12] and with the support of a consultant experienced in social research methodology who provided assistance in the selection, placement, response sets, content and face validity of the question items. In all questions on knowledge, opinion and attitude, a six-point Likert scale was used where 1 was negative and 6 positive. The survey instrument consisted of 32 questions with an estimated completion time of five to ten minutes. Six domains of questions were asked of respondents:

1. Student demographics and current tobacco use (defined as “use within the last 30 days”) and “ever tobacco use” (9 questions)

2. Knowledge and attitudes toward tobacco and waterpipe smoking (5 questions)

3. Health implications of smoking (3 questions)

4. Effectiveness and priority of tobacco cessation interventions (1 question)

5. Perceived education and competency (10 questions)

6. Perceived physicians role in tobacco cessation (4 questions).

### Procedures

The Health Ethics Review Board at the University of Alberta approved the survey. The Undergraduate Medical Education (UME) office posted a bulletin about the survey on the on-line learning community for the Faculty of Medicine and Dentistry that included a description of the survey, a link to the invitation letter and a link to the survey instrument. The UME office subsequently sent an email to all medical students alerting them to the bulletin. Students who read the bulletin and clicked on the link to the survey were taken to a secure internet site (FluidSurveys, Ottawa, ON, Canada) where they were asked to complete an anonymous informed consent form if they wished to participate in the survey. Potential participants were informed that for voluntarily completing the questionnaire, they were eligible to receive either a $5.00 unique OneCard certificate (the OneCard is the student identity card that can contain monetary value to purchase certain services and products on the university campus) or a $5.00 Starbucks e-gift card. Only those who wanted to receive the incentive were asked to provide their email address at the end of the questionnaire. The email addresses were separated from the survey data to maintain anonymity.

### Statistical analysis

Incomplete questionnaires were not used in the analysis. Demographic and “current use” and “ever use” data were summarized using descriptive statistics. Categorical variables were reported using frequencies, while continuous data were analyzed using means and standard deviations. All group comparisons for categorical variables were conducted using chi-square analyses where two-sided p-values <0.05 were considered statistically significant. Multivariate analysis was used to identify predictors for competency to counsel patients and prescribe medication. The Statistical Package for the Social Sciences (SPSS, Version 19.0, IBM, Armonk, NY, USA) was used for data management and statistical analyses.

## Results

### Response rate

We received 301 completed surveys and 12 incomplete responses out of 681 potential respondents. The survey response rate was calculated as:

(number of completes) / (number of completes + number of incompletes + number of refusals), which in this case was: (301) / (681) = 44.2%. The highest response rate (65%) was from students who had just completed their first year of school and the lowest (25%) from recent graduates.

### Sample characteristics

Of the 301 respondents, 51% were females and the mean age was 24.4 years (SD = 2.8). About 37% of respondents had just completed their first year of medical school, while 14% of respondents had just graduated from their fourth year (Table 1).

**Table 1:** Demographic characteristics of University of Alberta medical student respondents

	**n/N**	**%**
**Gender**		
*Female*	155/301	51.5
*Male*	146/301	48.5
**Class**		
*2015*	111/301	36.9
*2014*	77/301	25.6
*2013*	70/301	23.3
*2012*	43/301	14.3
**Smoking status**		
*Cigarettes (ever)*	90/301	29.9
*Cigarettes (current)*	10/301	3.3
*Cigars/Cigarillos (ever)*	122/301	40.5
*Cigars/Cigarillos (current)*	20/301	6.6
*Waterpipe (ever)*	120/301	39.9
*Waterpipe (current)*	18/301	6.0
	**Mean (SD)**	
**Age [years]**	24.4 (2.8)	21- 47

### Smoking prevalence and opinions

More students had ever smoked cigars/cigarillos (40.5%) or waterpipes (39.9%) than those who had ever smoked a cigarette (29.9%). Similarly, current smoking rates were higher for cigars/cigarillos (6.6%) and waterpipe (6.0%) than for cigarettes (3.3%).

Among those who had ever smoked a waterpipe, the majority (53.8%), had done so at a shisha/hookah café. The most common materials reported to be smoked in a waterpipe were tobacco (59.7%) and herbal products (42.9%). There was no difference in beliefs about the health consequences of waterpipe smoking between those who indicated that they were “ever” smokers and non-smokers. A significant minority of all respondents believed that smoking tobacco in a waterpipe was less harmful than smoking tobacco in the form of a cigarette. Surprisingly, 10% of those who had smoked a waterpipe indicated that they did not think that smoking tobacco in a waterpipe was addictive, a belief not shared by non-smokers (Table 2).

**Table 2:** Waterpipe smoking practices and beliefs among University of Alberta medical respondents

	**Waterpipe**						**Trend**	
	**Smokers**		**Non-smokers**		***χ2***	***p-value***	***χ2***	***p-value***
**Smoking location**	**n/N**	**%**	**n/N**	**%**				
*Shisha/Hookah Café*	64/119	53.8	NA					
*Home*	16/119	13.4	NA					
*Some other location*	39/119	32.8	NA					
**Smoking material**								
*Tobacco*	71/119	59.7	NA					
*Herbal products*	51/119	42.9	NA					
*Cannabis*	18/119	15.1	NA					
*Don’t know*	23/119	19.3	NA					
**Smoking tobacco in a waterpipe is less dangerous than smoking a cigarette**					3.97	0.55	1.75	0.18
*Strongly disagree*	27/119	22.7	48/181	26.5				
*Disagree*	37/119	31.1	56/181	30.9				
*Disagree somewhat*	19/119	16.0	32/181	17.7				
*Agree somewhat*	23/119	19.3	35/181	19.3				
*Agree*	10/119	8.4	9/181	5.0				
*Completely agree*	3/119	2.5	1/181	0.6				
**Smoking tobacco in a waterpipe is not addictive.**					21.48	< 0.001	12.4	<0.001
*Strongly disagree*	45/119	37.8	77/180	42.8				
*Disagree*	34/119	28.6	69/180	38.3				
*Disagree somewhat*	17/119	14.3	26/180	14.4				
*Agree somewhat*	10/119	8.4	7/180	3.9				
*Agree*	11/119	9.2	1/180	0.6				
*Completely agree*	2/119	1.7	0/180	0.0				
**Smoking herbal products in a waterpipe has no significant consequences to your health.**					2.69	0.75	0.44	0.5
*Strongly disagree*	40/120	33.3	60/180	33.3				
*Disagree*	44/120	36.7	76180	42.2				
*Disagree somewhat*	26/120	21.7	32/180	17.8				
*Agree somewhat*	6/120	5.0	8/180	4.4				
*Agree*	4/120	3.3	3/180	1.7				
*Completely agree*	0/120	0.0	1/180	0.6				

### Perception of exposure to tobacco related education

**Figure 1:**
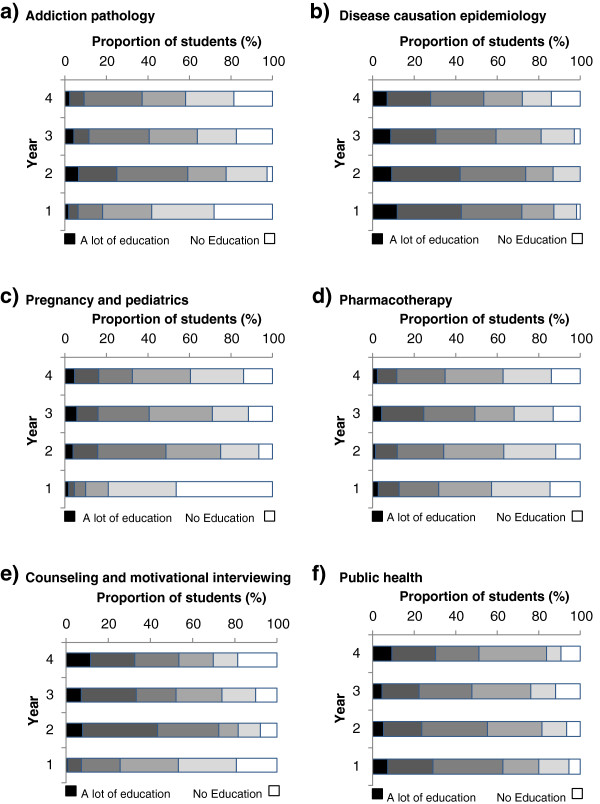
**Perceived amount of tobacco education received in each year of medical school regarding six special areas.** Students from each year were asked to rate the amount of tobacco education they received in the past year on a scale of 1 to 6 with “1” being “no education” and “6” being “a lot of education” for each of the six special areas (panels **a** to **f**). Student responses were stratified according to their year of medical education from 1-4.

Respondents reported moderate levels of education on a variety of tobacco-related subjects. The overall tobacco education score was 3.25 (95% CI 3.14-3.36). Limited education was reported on the role of tobacco in addiction pathology, pregnancy and pediatrics and on the pharmacotherapy of nicotine addiction. Respondents reported greater exposure to education about counseling and motivational interviewing techniques and the role of tobacco in public health and the most education about the role of tobacco causing in disease. Less than 10% of respondents reported that they received “a lot of education” about any of the subject areas surveyed (Figure 1).

### Perceived competence in tobacco cessation interventions

**Figure 2:**
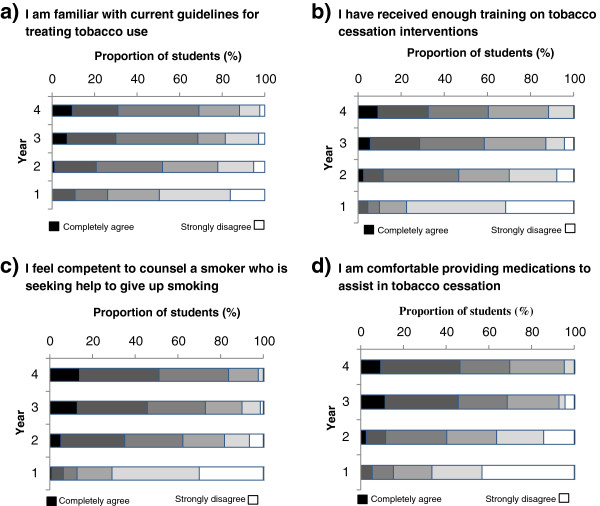
**Self-reported competencies of medical students regarding tobacco cessation interventions.** Students were asked to rate their level of agreement with the above statements. The rating was on a scale of 1 to 6 with “1” being “strongly disagree” and “6” being “completely agree” for each of the four tobacco cessation interventions (panels **a** to **d**). Student responses were stratified according to their year of medical education from 1-4.

Fewer than 10% of respondents in any year, *strongly agreed* with the statements “I have received enough training on tobacco cessation interventions”; “I am comfortable providing medications to assist in tobacco cessation” and “I am familiar with the current guidelines for treating tobacco use”, however, some 30% of final-year students felt knowledgeable (*strongly agree* and *agree somewhat*) about guidelines and were satisfied with the education they have received. Fifty per cent felt competent to counsel patients and prescribe medication. A difference in self-reported competence was apparent in the responses as students progressed through the program (Figure 2). Multivariate analysis showed that the year of education was the only significant factor in predicting if students were likely to counsel smokers who are seeking help to give up smoking.

### Health consequences of smoking

**Figure 3:**
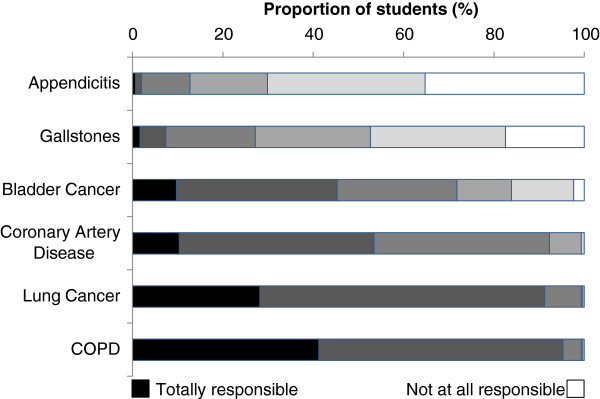
**The responsibility of cigarettes for six diseases.** Diseases were given in a randomized order and the students were asked to rate the degrees to which cigarettes are responsible for each disease. The rating was on a scale of 1 to 6 with “1” being “not at all responsible” and “6” being “totally responsible”. COPD, chronic obstructive pulmonary disease.

Most students believed that cigarettes are either *mostly or totally responsible* for chronic obstructive pulmonary disease (95%), lung cancer (91%), coronary artery disease (54%) and, to a lesser extent, bladder cancer (Figure 3). Most students thought that cigarettes were *not at all* or *not very responsible* for the development of gallstones and appendicitis. The majority of students (92%) agreed that non-smokers could expect to live an average of ten years longer than smokers; however, they did not believe that quitting at the age of 30 would extend a smoker’s life expectancy to that of a life-long non-smoker.

### Tobacco cessation

**Figure 4:**
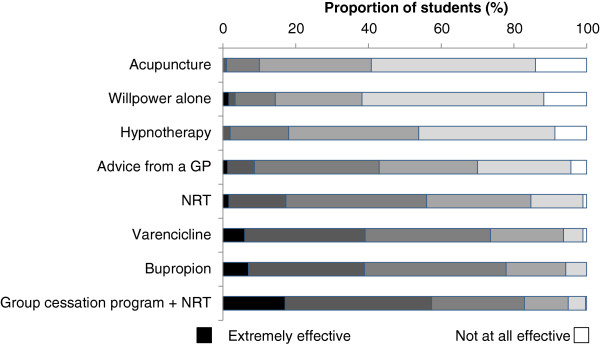
**Perceived effectiveness of smoking cessation methods.** Methods were given in a randomized order and the students were asked to rate the effectiveness of each method on a scale of 1 to 6 with “1” being “not at all effective” and “6” being “extremely effective”. NRT, nicotine replacement therapy; GP, general practitioner.

Most participants believed that pharmacotherapies alone were at least somewhat effective in tobacco cessation (Figure 4). The intervention most regarded as *extremely effective* by 17.1% of students was “a group cessation program including several sessions and nicotine replacement therapy”. Most participants thought that both advice from a general practitioner and willpower alone were *somewhat ineffective*. Respondents generally agreed (85%) that physicians play a leadership role in tobacco management and that it is a priority for physicians. However, nearly one-third of students did not think that physicians are motivated to implement tobacco cessation programs with their patients and 35% of respondents did not believe that tobacco cessation counseling is a priority in Alberta.

## Discussion

This survey was carried out to assess the use of tobacco products and the tobacco-related attitudes, knowledge, and education of University of Alberta medical students. Self-reported cigarette use among our survey population was low, but lifetime exposure to alternative methods of tobacco consumption (waterpipe, cigars) was surprisingly common. While students reported some exposure to all domains of tobacco education surveyed, they expressed only moderate confidence in their own ability to counsel and treat smokers. This was despite correctly identifying the key role of tobacco in the pathogenesis of lung cancer, chronic obstructive pulmonary disease and coronary artery disease.

Self-reported current cigarette use among our study population was low (3.3%). In contrast, recent surveys of medical students in Germany, the USA and the UK reported much higher rates of cigarette use (20%, 11% and 10%, respectively) [11-13]. The only other recent survey of cigarette use among Canadian medical students reported similar data to that reported in this study [9]. The latest WHO estimates for adult cigarette smoking prevalence were 29% in Germany, 24% in the United Kingdom, 26% in the United States, and 17% in Canada [14]. Although self-reported cigarette use among medical students in Canada is lower than the use of cigarettes among the general population, it is concerning that current cigar/cigarillo use among this health aware group is equivalent to that in the general population and exceeds it in the case of the use of the waterpipe [15]. Although 40% of respondents indicated that they had smoked a waterpipe at some point, they were skeptical of the alleged safety of this practice. Several studies confirm their suspicions, asserting that waterpipe smoking is both harmful and addictive; smokers are exposed to high levels of particulate matters, carbon monoxide, and other chemicals, which affect lung health [16]. More than half of those who had ever smoked a waterpipe did so in a public café. The proportion of students being introduced to waterpipe in these venues highlights the importance of smoke-free legislation being applied to waterpipe cafes [17]. Almost half of the students who ever smoked the waterpipe did so at home or in other locations, suggesting that we may be witnessing a normalization of waterpipe smoking in Canada among young adults. Most respondents indicated that they chose to smoke tobacco in their waterpipes, a practice that carries with it the real risk of nicotine addiction [18].

While the causative role of tobacco in the etiology of disease was well known by respondents, they indicated that they received less education about the management of tobacco use in pregnancy and childhood. This may well be a reflection of the relative paucity of research and the difficulty in treating tobacco addiction in both these groups. Tobacco use during pregnancy is a key modifiable risk factor for innumerable perinatal complications but poor implementation and psychosocial barriers frequently compromise the success of cessation efforts [19]. Nevertheless, it is vital that physicians champion smoking cessation interventions as a routine part of prenatal care, as the long term benefits to both child and mother are inestimable. The adverse consequences of second hand smoke on child health have been well described and the imperative for treatment of nicotine addiction compelling [20]. The results of our survey suggest that a focused tobacco management module in both the pediatric and obstetric blocks could well be appropriate with an emphasis on motivational techniques to promote behavior change.

Less than 10% of graduating respondents *strongly agreed* that they were familiar with the current guidelines for treating tobacco use, in comparison, 99% of general practitioners are familiar with hypertension guidelines and 90% of clinicians are familiar with the guidelines for the treatment of angina [21,22].

These data point to the need to encourage widespread exposure of medical students in Canada to the excellent CAN-ADAPTT resource which includes not only guideline information but also links to a range of educational resources including instruction of motivational interviewing techniques [23].

It was disappointing that although a role for leadership by physicians in tobacco addiction management was acknowledged, one-third of respondents believed that physicians were not motivated to implement tobacco cessation programs and more than half thought that their advice would be ineffective. These data mirrored findings in a Quebec study which demonstrated that while many physicians discussed tobacco addiction with their patients in two years of seeing them [24], a significant minority perceived that these interventions took too much time or were not effective [25]. As knowledge-based learning is cultivated and reinforced through clinical observation, it is important that practicing physicians model the appropriate attitudes and actions towards tobacco use [26]. Although medical students from different countries agreed on the dangers of tobacco use, they differed in their perception of effective cessation methods. While medical students in London and Edmonton rated pharmacotherapy as more effective than physician advice, willpower or alternative therapy, and perceived “group cessation programs including nicotine replacement therapy (NRT)” to be the most effective tobacco cessation intervention, German medical students viewed “willpower alone” to be extremely effective [11]. Though advice from a physician doubles a smoker’s chances of quitting [27], the majority of students from medical schools in Canada, Britain and Germany viewed “GP advice” as *ineffective*[11]. Despite their confidence in counseling and medicating tobacco users, this view may discourage clinical students at the University of Alberta from offering appropriate advice to current smokers.

### Limitations

A self-administered survey is a convenient and effective way to measure knowledge and opinions but has inherent drawbacks. Our survey was only able to measure education and competency subjectively, relying on students to report on these parameters accurately. Objective measures of clinical competency must be sought through other modalities.

Within these constraints, we took steps to ensure that the data we did obtain were of optimal quality by developing clear and concise wording and placement of question items and providing a logical flow to reduce respondent bias, burden and the length of the survey instrument. *Heerwegh and Looseveldt*[28] found that, when comparing data quality in face-to-face versus on-line survey responses, those who responded on a web survey were more inclined to opt for “don’t know” responses. To minimize this effect, our survey instrument contained only one item with a “don’t know” response category. The use of on-line surveys has been found to increase reporting of sensitive issues (like smoking behavior), increase the accuracy of reporting and decrease the likelihood of a question being skipped compared to other methods of data collection, especially among young, well-educated and computer literate populations [29].

A response rate of 44.2% is well within an acceptable range for online surveys of medical students. For example, a one-week survey of New York medical students resulted in a 30% response rate [30]; a 25-week mixed mode survey of Canadian physicians and medical students produced an email response rate of 29.9% [31] and an on-line survey of fourth-year medical students enrolled in six schools in New York City, which ran for 13.5 weeks, garnered a response rate of 50% [12]. Response rates were not uniform across the University of Alberta medical classes. Conclusions, especially those concerning the class of 2012, must be interpreted with caution. Our overall data suggest that there may be room for improvement in tobacco cessation intervention training. As the sampling frame was limited to one university, it may not be representative of medical education in all of Canada. Nonetheless, given the limited numbers of medical schools in Canada and the standardization of medical curriculum through licensing exams, we can assume that some of these findings may be relevant to other Canadian institutions.

## Conclusions

University of Alberta medical students have lower cigarette smoking rates than students in Europe but seem to use cigars and waterpipes quite frequently. They are knowledgeable about the health consequences of tobacco addiction but still require education on the practical aspects of the treatment of tobacco addiction.

### Practice implications

Although cigarettes have been the main focus of tobacco education, students and physicians must not overlook other forms of tobacco use such as cigars, cigarillos or waterpipes. Further analysis of both student and physician awareness of alternative tobacco use will inform continuing education on the subject and updated guidelines for the treatment of tobacco use. Additional training on behavior modification therapy and the effect of physician advice on patient behavior is also needed to address an overemphasis on pharmacotherapy. As medical students learn from a combination of didactic lectures, interactive exercises, and mentor role modeling, regular practice of preventative behavioral change interventions amongst physician preceptors can teach future physicians to employ this method as well. An evaluation of recent graduates in clinical practice will provide further clues into the long-term effectiveness of current tobacco medical education. Medical students and current physicians would benefit from the tools, support and ongoing education to make them effective leaders in tobacco cessation and health promotion.

## Competing interests

TR has received honoraria from Pfizer®, Novartis®, Glaxo Smith Kline®, Astra Zeneca® and Roche® as a speaker in activities related to continuing medical education. All other authors have none to declare.

## Authors’ contributions

AV carried out the survey, collected and analyzed data, and drafted the article with input from other authors. FH was instrumental in survey development, drafted the introduction and methods for ethics review, assisted in data analysis and provided critical revisions to the article. AC contributed to survey development, submitted the study for ethics approval, directed survey implementation and contributed to the methods section. CW assisted in question design and data interpretation. TR assisted in the design of the study, interpretation of the results and contributed to the discussion of the findings. BF is the principal investigator, responsible for the study conception, design, data interpretation and editorial revisions. All authors read and approved the final manuscript.
